# New insights about immune populations in gastrointestinal GvHD

**DOI:** 10.1016/j.xcrm.2023.101126

**Published:** 2023-07-18

**Authors:** Eiko Hayase, Robert R. Jenq

**Affiliations:** 1 Department of Genomic Medicine, The University of Texas MD Anderson Cancer Center, Houston, TX 77054, USA; 2 Department of Stem Cell Transplantation and Cellular Therapy, The University of Texas MD Anderson Cancer Center, Houston, TX 77030, USA

## Abstract

Jarosch et al.^1^ have deeply characterized immune cell infiltrates in gastrointestinal (GI) biopsies from individuals with GI graft-versus-host disease (GI-GvHD) using single-cell RNA sequencing and ChipCytometry. Individuals with severe GI-GvHD demonstrated increased clonally expanded cytotoxic CD8 T cells in GI biopsies.

## Teaser

Jarosch et al. have deeply characterized immune cell infiltrates in gastrointestinal (GI) biopsies from individuals with GI graft-versus-host disease (GI-GvHD) using single-cell RNA sequencing and ChipCytometry. Individuals with severe GI-GvHD demonstrated increased clonally expanded cytotoxic CD8 T cells in GI biopsies.

## Main text

Graft-versus-host disease (GvHD) is an alloreactive T cell-mediated systemic inflammatory disease and remains a major obstacle in allogeneic hematopoietic stem cell transplantation (aHSCT). The gastrointestinal (GI) tract is often targeted by GvHD, and severe GI-GvHD tends to have a poor prognosis.^2^ Use of antibiotics is essential for aHSCT individuals because of the high risks of bacterial infections but can also disrupt the intestinal microbiota.^3^ The intestinal microbiota is an important modulator of intestinal GvHD,^4^ and microbiota injury has been strongly associated with acute GvHD and reduced overall survival.^5^ However, it is not fully understood what factors that determine GI-GvHD severity interact with the intestinal microbiota.

Here, Jarosch and colleagues^1^ collected a multifaceted dataset including single-cell RNA sequencing (scRNA-seq) of GI biopsies, GI tissue imaging using ChipCytometry, and fecal microbiome analysis using 16S ribosomal RNA gene sequencing (16S rRNA-seq) collected at the same time. In this study, the authors began by obtaining biopsy samples from aHSCT individuals at three time points: screening, onset of GvHD, and more than 1 week following onset. Biopsies were classified as mild (stage 0–1) or severe (stage 2–4) cases of GvHD. For comparison purposes, non-inflamed adjacent tissue samples from colon resections of individuals with colorectal cancer were used.

Evaluating infiltrating immune cell populations using scRNA-seq, the authors observed in individuals with aHSCT compared to control cases increases in monocytes, CD4 memory cells, and CD8-activated T cells, as well as reductions in B cells, plasma cells, and mast cells. Among aHSCT individuals, CD8-activated T cells and regulatory CD4 T cells (Tregs) were increased in samples from individuals with severe GvHD compared to those with mild GvHD. To complement these findings, the authors employed ChipCytometry, a multi-parameter tissue section imaging method, and found that severe GvHD samples indeed showed significantly more infiltration of CD8 T cells compared to mild GvHD samples although there was no clear increase in Treg infiltration. T cell receptor (TCR) repertoire analysis showed that this expansion of CD8 T cells was clonal in aHSCT individuals compared to control cases. The degree of increased clonality and consequently decreased TCR diversity were significantly associated with GvHD severity. Importantly, these findings were observed at multiple time points after aHSCT.

Immune cell phenotypes of GI biopsies in aHSCT individuals are heterogeneous because of complex clinical backgrounds. It is necessary to carefully consider the potential for confounding effects of the state of immune reconstitution when analyzing biopsies from different time points. Another potential modulator of immune cell phenotypes from GI biopsies includes the use of antibiotics, which leads to dynamic alterations of the microbiota composition. To investigate these factors, the authors grouped affected individuals into antibiotic-exposed and non-antibiotic-exposed groups. In the non-antibiotic-exposed group, severe GvHD cases showed a trend toward higher Treg infiltration compared to mild GvHD cases. Interestingly, this association was not observed in the antibiotic-exposed group, suggesting that Treg infiltration was affected by exposure to antibiotics or subsequent alterations to the microbiota composition.

Importantly, the authors identified three distinct Treg clusters by examining expression of *FOXP3*, a hallmark of Tregs. One of them showed significantly higher gene signatures associated with Treg functions, whereas others showed an effector T cell-like signature, indicating reduced Treg functionality. The authors next investigated whether specific bacterial taxa were linked to differentiation of functional Tregs using 16S rRNA-seq. They found that the classes Clostridia and Bacteroidia were associated with functional Treg differentiation. Intestinal Clostridia and Bacteroidia are the primary producers of short-chain fatty acids, including butyrate and propionate, which are known to introduce Tregs in the GI tract,^6^^,^^7^ suggesting the potential for an effect of the microbiome composition on Treg induction in aHSCT individuals.

Finally, to investigate how therapeutic interventions targeting the microbiome composition could act on the Treg/CD8 T cell balance, the authors evaluated GI biopsies collected from one individual with severe GvHD before and after undergoing fecal microbiota transplantation (FMT). This individual experienced a significant increase in microbial diversity and complete response of GI-GvHD at 9 months of follow-up. Interestingly, treatment with FMT led to a trend of decreased CD8 T cell infiltration along with increased Treg infiltration. While results of a single case study are not definitive, these findings suggest that the immunosuppressive potential of Tregs can be tuned by microbiota composition and altered abundances of Tregs as well Treg functionality and suppression of CD8 T cells.

scRNA-seq is a next-generation sequencing method that enables unbiased and high-resolution genome-wide assessment of individual cells. This technique is becoming the gold standard for investigating cell heterogeneity. However, scRNA-seq for analysis of biopies from individuals with GI-GvHD has been understudied thus far. One previous study employing scRNA-seq in a murine model of GvHD identified an intestinal CD4^+^ GM-CSF^+^ T cell subset that contributes to pathologic damage in the GI tract.^8^ Another study, in a non-human primate GvHD model, utilized intravascular staining combined with scRNA-seq to demonstrate that donor-derived CD8 T cells infiltrating into the GI tract were highly activated and cytotoxic and that levels of CD8 T cell infiltration into the GI tract were positively correlated with severity of GvHD,^9^ consistent with the current study. The TCR repertoire is shaped by exposure of various factors such as infection and alloantigens in aHSCT. Because the TCR repertoire is highly dynamic in the early post-aHSCT setting as the immune system recovers, characterizing the TCR repertoire may allow prediction of clinical outcomes or toxicities.^10^ Thus, a combination of these modalities could allow us to find new insights for understanding and managing GI-GvHD.

In conclusion, this study has uncovered new insights regarding GI tissue infiltrating immune cell populations in individuals with GvHD utilizing a combination of cutting-edge technologies. Clonally expanded CD8-activated T cells in the GI tract appear to be important mediators of severe GI-GvHD. However, how clonally expanded CD8 T cells induce severe GI-GvHD and how Tregs are linked to CD8 T cells are still unclear. In addition, the impact of the microbiota or microbe-derived metabolites on GvHD severity has not yet been completely explored. To elucidate these points, further studies will be necessary, including large prospective cohort studies and well-designed animal models.
